# Long non-coding RNA BCYRN1 exerts an oncogenic role in colorectal cancer by regulating the miR-204-3p/KRAS axis

**DOI:** 10.1186/s12935-020-01543-x

**Published:** 2020-09-14

**Authors:** Liu Yang, Yinan Zhang, Jun Bao, Ji-Feng Feng

**Affiliations:** 1 Department of Colorectal Surgery, The Affiliated Cancer Hospital of Nanjing Medical University & Jiangsu Cancer Hospital & Jiangsu Institute of Cancer Research, Nanjing, China; 2 Department of Chemotherapy, The Affiliated Cancer Hospital of Nanjing Medical University & Jiangsu Cancer Hospital & Jiangsu Institute of Cancer Research, No. 42 Baiziting, Nanjing, China

**Keywords:** lncRNA BCYRN1, miR-204-3p, KRAS, Colorectal cancer

## Abstract

**Background:**

It has been well documented that long non-coding RNAs (lncRNAs) regulate numerous characteristics of cancer, including proliferation, migration, metastasis, apoptosis, and even metabolism. LncRNA BCYRN1 (BCYRN1) is a newly identified brain cytoplasmic lncRNA with 200 nucleotides that was discovered to be highly expressed in tumour tissues, including those of hepatocellular carcinoma, gastric cancer and lung cancer. However, the roles of BCYRN1 in colorectal cancer (CRC) remain obscure. This study was designed to reveal the role of BCYRN1 in the occurrence and progression of CRC.

**Methods:**

RT-PCR was used to detect the expression level of BCYRN1 in tumour tissues and CRC cell lines. BCYRN1 was knocked down in CRC cells, and cell proliferation changes were evaluated by cell counting kit-8 (CCK-8), 5-ethynyl-2′-deoxyuridine (EdU), and Ki-67 and proliferating cell nuclear antigen (PCNA) expression assays. Cell migration and invasion changes were evaluated by wound healing, Transwell and invasion-related protein expression assays. Flow cytometry analysis was used to assess whether BCYRN1 regulates the apoptosis of CRC cells. The dual luciferase reporter gene detects the competitive binding of BCYRN1 to miR-204-3p. In vivo experiments were performed to evaluate the effect of BCYRN1 on tumour development. TargetScan analysis and dual luciferase reporter gene assays were applied to detect the target gene of miR-204-3p. Rescue experiments verified that BCYRN1 affects CRC by regulating the effect of miR-204-3p on KRAS.

**Results:**

We found that compared with normal tissues and human intestinal epithelial cells (HIECs), CRC tumour tissues and cell lines had significantly increased BCYRN1 levels. We further determined that knockdown of BCYRN1 inhibited the proliferation, migration, and invasion and promoted the apoptosis of CRC cells. In addition, bioinformatics analysis and dual luciferase reporter assay showed that BCYRN1 served as a competitive endogenous RNA (ceRNA) to regulate the development of CRC through competitively binding to miR-204-3p. Further studies proved that overexpression of miR-204-3p reversed the effects of BCYRN1 on CRC. Next, TargetScan analysis and dual luciferase reporter assay indicated that KRAS is a target gene of miR-204-3p and is negatively regulated by miR-204-3p. A series of rescue experiments showed that BCYRN1 affected the occurrence and development of CRC by regulating the effects of miR-204-3p on KRAS. In addition, tumorigenesis experiments in a CRC mouse model confirmed that BCYRN1 downregulation effectively inhibited tumour growth.

**Conclusions:**

Our findings suggest that BCYRN1 plays a carcinogenic role in CRC by regulating the miR-204-3p/KRAS axis.

## Background

Colorectal cancer (CRC) has a high incidence and mortality worldwide; specifically, it ranks third in cancer morbidity and second in cancer mortality globally [1, 2]. CRC has become one of the most common and dangerous malignancies in China [3]. The mainstay treatment for CRC is surgery, chemotherapy, radiotherapy and other comprehensive therapies, but the 5-year survival rate has not changed significantly in recent decades and is still poor [4, 5]. Therefore, delivering high-efficiency CRC diagnosis and treatment methods should be the aim of all physicians in internal medicine research. The present study aims to provide an up-to-date molecular mechanism of CRC occurrence.

Recently, epigenetic changes have been reported to play important roles in the occurrence and progression of malignancies [6, 7]. LncRNAs are a class of transcripts with lengths greater than 200 nucleotides and have limited or non-protein coding potential [8–10]. Various studies have demonstrated that lncRNAs participate in the multilevel regulation of gene expression by targeting microRNAs (miRNAs), mRNAs, and even proteins [11, 12]. Furthermore, growing evidence has proven that lncRNAs regulate multiple characteristics of cancer, such as proliferation, migration, apoptosis, and even metastasis [13, 14]. BCYRN1 is a brain cytoplasmic lncRNA that is newly identified and activated by c-MYC [15]. It has been reported that BCYRN1 is highly expressed in various tumour tissues [16], such as hepatocellular carcinoma [17], gastric cancer [18], and lung cancer [15] but is expressed at lower levels in healthy control (HC) tissues. In addition, BCYRN1 plays an important role in inhibiting smooth muscle differentiation and vascular development in the cardiovascular system [19, 20]. However, little is known about the role of BCYRN1 in CRC, which aroused our great interest.

MiRNAs are a group of small, non-coding RNAs with nucleotides of approximately 22 and are identified as negative regulators [21, 22]. miRNAs can act as targets of lncRNAs and participate in the regulation of gene expression. Specifically, miRNA is involved in gene expression by binding to the complementary sequence location of the 3′-untranslated region (3′-UTR) of target mRNA to disturb the structural stability and translation of mRNAs [23, 24]. Increasing evidence has demonstrated that miRNAs play crucial roles in cancer, cardiovascular disease and other diseases [22, 25] by regulating relative cell proliferation, migration, invasion and apoptosis [23]. On the other hand, lncRNAs competitively bind to miRNAs as ceRNAs, resulting in the inability of the miRNAs to combine with their corresponding mRNAs, thus regulating gene expression and function [11, 26, 27].

The present study aimed to determine the function and underlying mechanism of BCYRN1 in CRC progression. Our research determined that BCYRN1 plays an oncogenic role in CRC by regulating the miR-204-3p/KRAS axis, revealing a novel mechanism for the occurrence and development of CRC and providing a novel therapeutic and prognostic target for CRC.

## Materials and methods

### Experimental human tissues and animals

The research tissues of CRC patients and healthy controls were obtained from The Affiliated Cancer Hospital of Nanjing Medical University, and all patients signed written consent forms. Tissue debris was immediately frozen in liquid nitrogen after surgery and stored at − 80 °C. All animal experiments were designed according to the standards of the Guide for the Care and Use of Laboratory Animals (NIH, 8th edition, 2011). The Ethics Committee of The Affiliated Cancer Hospital of Nanjing Medical University approved the present research.

### Cell culture

Four human CRC cell lines, LoVo, HCT116, SW480, and SW620 and HIECs, were purchased from American Type Culture Collection (ATCC; Manassas, VA, USA) and cultured in DMEM (Gibco, Grand Island, NY, USA) containing 10% foetal bovine serum (FBS; Gibco, Grand Island, NY, USA), 100 IU/mL penicillin and 1 × 10^5^ μg/mL streptomycin in a humidified atmosphere containing 5% CO_2_ at 37 °C.

### Cell transfection

shRNA-BCYRN1 (sh-BCYRN1), shRNA-KRAS (sh-KRAS) and corresponding shRNA negative control (sh-NC) transfection were performed using Lipofectamine 3000 reagent (Invitrogen, Carlsbad, CA, USA) according to the manufacturer’s instructions. In addition, RNAifectin™ transfection reagent, NC, miR-204-3p mimic and miR-204-3p inhibitor were obtained from Applied Biological Materials Inc. (Richmond, BC, Canada). HIECs and CRC cells were cultured in 6-well plates for transfection, and the efficiency of transfection was determined by qRT-PCR.

### RNA extraction and qRT-PCR

Total RNA was extracted from human tissues, mouse tissues and cells with TRIzol reagent (Life Technologies, Gaithersburg, MD, USA). Reverse transcription experiments were performed with PrimeScript® RT reagent Kits (Takara, Otsu, Shiga, Japan) and a StepOnePlus™ Real-Time PCR System (Applied Biosystems, Foster City, CA, USA). Next, quantitative reactions were performed with SYBR Green RT-PCR (Takara Biotechnology Co., Ltd., Tokyo, Japan) using the StepOnePlus™ system. GAPDH and U6 snRNA were used as the internal controls.

### Evaluation of cell proliferation

Cell proliferation was detected by cell counting kit-8 (CCK-8) assay, 5-ethynyl-2′-deoxyuridine (EdU) incorporation test, and nucleoprotein Ki-67 and proliferating cell nuclear antigen (PCNA) expression analysis. First, for the CCK-8 experiment, cells were seeded into a 96-well plate with 10% FBS DMEM for 24 h. Then, the medium was changed with serum-free DMEM for CCK-8 kit (Beyotime Biotechnology, Shanghai, China) detection. A microplate reader (Model ELX800, BioTek, Vermont, USA) was used to determine the absorbance at 450 nm, which reflects cell viability and proliferation. Second, cells were seeded into a 24-well plate for EdU assessment. After 48 h of transfection, DNA synthesis was examined with an EdU incorporation assay (Guangzhou RiboBio, Guangzhou, China). The proportion of EdU-positive cells was analysed by fluorescence microscopy (DP70, Olympus Optical, Tokyo, Japan). Finally, cell proliferation was evaluated by Ki-67 and PCNA expression. Ki-67 is a nucleoprotein that is a marker of tumour proliferation [28]. PCNA acts on chromatin and participates in all aspects of the DNA replication chain [25]. The expression levels of Ki-67 and PCNA can be used to evaluate the status of cell proliferation.

### Evaluation of cell migration and invasion

Cell migration and invasion were assessed with the wound healing assay, Transwell assay and invasion-related protein expression. For the wound healing assay, cells were seeded into a 6-well plate. A sterile 1-mL pipette tip was used to scratch the bottom of the plate to form a gap. The images of cell migration were captured at 0 h and 48 h after transfection, respectively, with an inverted microscope (Axio Vert. A1, Zeiss, Oberkochen, Germany). The scratch distance at 48 h was subtracted from the scratch distance at 0 h to calculate the percentage closure of the wound [29]. The average distance of migration reflects the cell migration capacity. For the Transwell assay, cells were plated into the no FBS medium in the upper chamber of a 12-well Transwell with an 8-μm pore size (Merck kGaA, Darmstadt, Germany). FBS medium (10%) was added to the lower chamber. After 24 h, the cells that migrated to the submembrane surface were stained with crystal violet. Stained cells were counted in 6 randomly selected regions.

### Flow cytometric analysis

Flow cytometric analysis was designed to evaluate whether BCYRN1 regulated the apoptosis of CRC cells. Consistently, cells were seeded into a 6-well plate. The cells were collected and stained with annexin V-FITC (Beyotime Biotechnology, Shanghai, China) and propidium iodide (PI) reagent after 48 h transfection according to the manufacturer’s instructions. Then, cell apoptosis was determined by flow cytometry (BD FACSCalibur cytometer, Becton Dickinson, San Jose, CA, USA).

### Dual luciferase reporter assay

The partial sequences of BCYRN1 and the 3′-untranslated region (3′-UTR) of KRAS containing the putative binding sites of miR-204-3p were synthesized and inserted into a luciferase reporter vector plasmid (GenePharma, Shanghai, China). According to the instructions, firefly luciferase reporter plasmids and equal amounts of miR-204-3p mimics or NC mimics were transfected into cells. Then, the relative luciferase activities were detected by the Dual-Luciferase Reporter Assay System (Promega, Madison, WI, USA) on a Luminometer 20/20n (Turmer Biosystems, Sunnyvale, CA, USA) after transfection for 48 h. Renilla luciferase activity was employed as an internal control for cellular density and transfection efficiency.

### In vivo experiments

The nude mice were injected subcutaneously with CRC cells (approximately 10^6^ cells) into the right flanks. Then, sh-NC or sh-BCYRN1 was directly injected into the mice to knockdown BCYRN1. Tumour size was measured every 5 days for a total of 6 measurements. At the end of the experiments, mice were humanely sacrificed with overdose anaesthesia for the collection of tumours. Each tumour was weighed to evaluate the effects of BCYRN1 on the development of tumours.

### Immunohistochemistry

Tumours were obtained, fixed in 4% paraformaldehyde, embedded in paraffin, and then cut into 4 μm sections. After deparaffinization and blocking, the sections were incubated with primary anti-Ki-67 (1:100; Abcam; Cambridge, MA, USA) overnight at 4 °C. The immunofluorescence images were captured with an Olympus BX51 microscope (Olympus, Tokyo, Japan) after incubating horseradish peroxidase-conjugated goat anti-rabbit antibody.

### HE staining and TUNEL staining

Haematoxylin–eosin (HE) staining and terminal deoxynucleotidyl transferase (TdT)-mediated dUTP nick-end labelling (TUNEL) staining were performed to detect cancer cell apoptosis in mouse tumour tissues. Briefly, mouse tumour tissues were prefixed, and the paraffin-embedded sections were stained with haematoxylin–eosin and TUNEL (containing 2 μL TdT enzyme, 48 μL Fluorescent labelling solution and 50 μL TUNEL detection solution) according to the manufacturer’s instructions. The images were obtained with an Olympus BX51 microscope (Olympus, Tokyo, Japan) coupled with an Olympus DP70 digital camera.

### Western blot analysis

Cyclooxygenase-2 (Cox-2) is an enzyme complex that plays significant roles in metastasis and invasion of malignancies [30]. The matrix metalloproteinase (MMP) family, especially the gelatinases MMP-2 and MMP-9, are recognized as markers of tumour migration and invasion [31]. The expression of Cox-2, MMP-2 and MMP-9 can reflect the status of cell migration and invasion. The protein expression levels of Ki-67, PCNA, Cox-2, MMP-2, MMP-9, Bax, Bcl-2, cleaved caspase-3, cleaved caspase-9 and KRAS were detected by Western blot analysis. Total protein was extracted, and equal amounts of protein (30 μg) were separated by SDS-PAGE and then transferred to PVDF membranes. Protein bands were visualized with the Enhanced Chemiluminescence Detection Kit (Thermo Fisher Scientific, Rockford, IL, USA). All primary antibodies were obtained from Abcam (Cambridge, MA, USA). The dilution of PCNA antibody was 1:5,000, and the dilution ratio of other primary antibodies was 1:1,000.

### Statistical analysis

Experiments were conducted in a randomized, double-blinded situation. Data are expressed as the mean ± SD. Student’s unpaired t-test was used to compare the differences between two groups. One-way or two-way ANOVA followed by post hoc Bonferroni test was employed for multiple comparisons. Statistical significance was considered as *P < 0.05, **P < 0.01, and ***P < 0.001.

## Results

### BCYRN1 is highly expressed in the tissues and cell lines of CRC

**Fig. 1:**
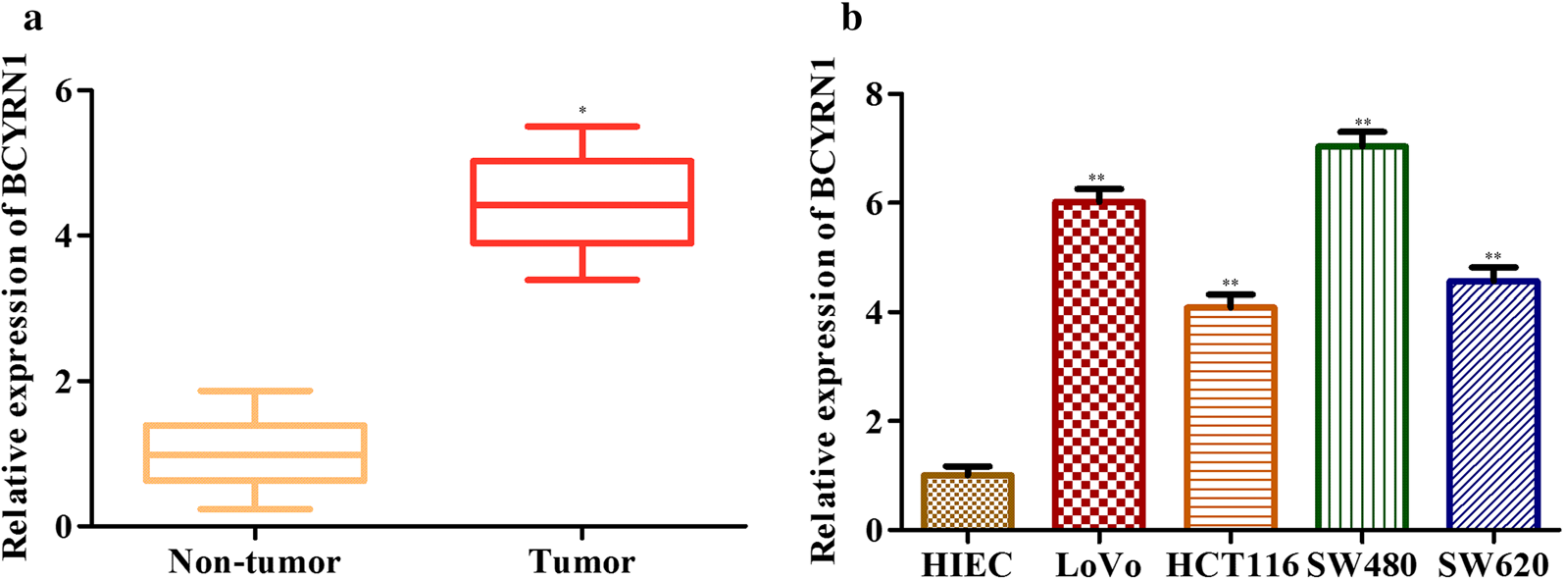
LncRNA BCYRN1 is highly expressed in the tissues and cell lines of CRC. **a** BCYRN1 levels in HC tissues and CRC tumour tissues. Data from 15 HC people and 15 GC patients. **b** BCYRN1 levels in HIECs and CRC cell lines. Values are the mean ± SD. *P < 0.05, **P < 0.01, and ***P < 0.001 vs. HCs or HIECs. n = 3 per group

In our preliminary study, we noted that BCYRN1 was highly expressed in CRC tissues (Fig. 1a). Then, we examined the expression levels of BCYRN1 in HIECs and four types of human CRC cell lines. Consistently, BCYRN1 expression was significantly increased in CRC cell lines (Fig. 1b). These data aroused our interest, as they suggested that BCYRN1 levels may be closely related to the development of CRC.

### Knockdown of BCYRN1 inhibits the proliferation and promotes the apoptosis of CRC cells

**Fig. 2:**
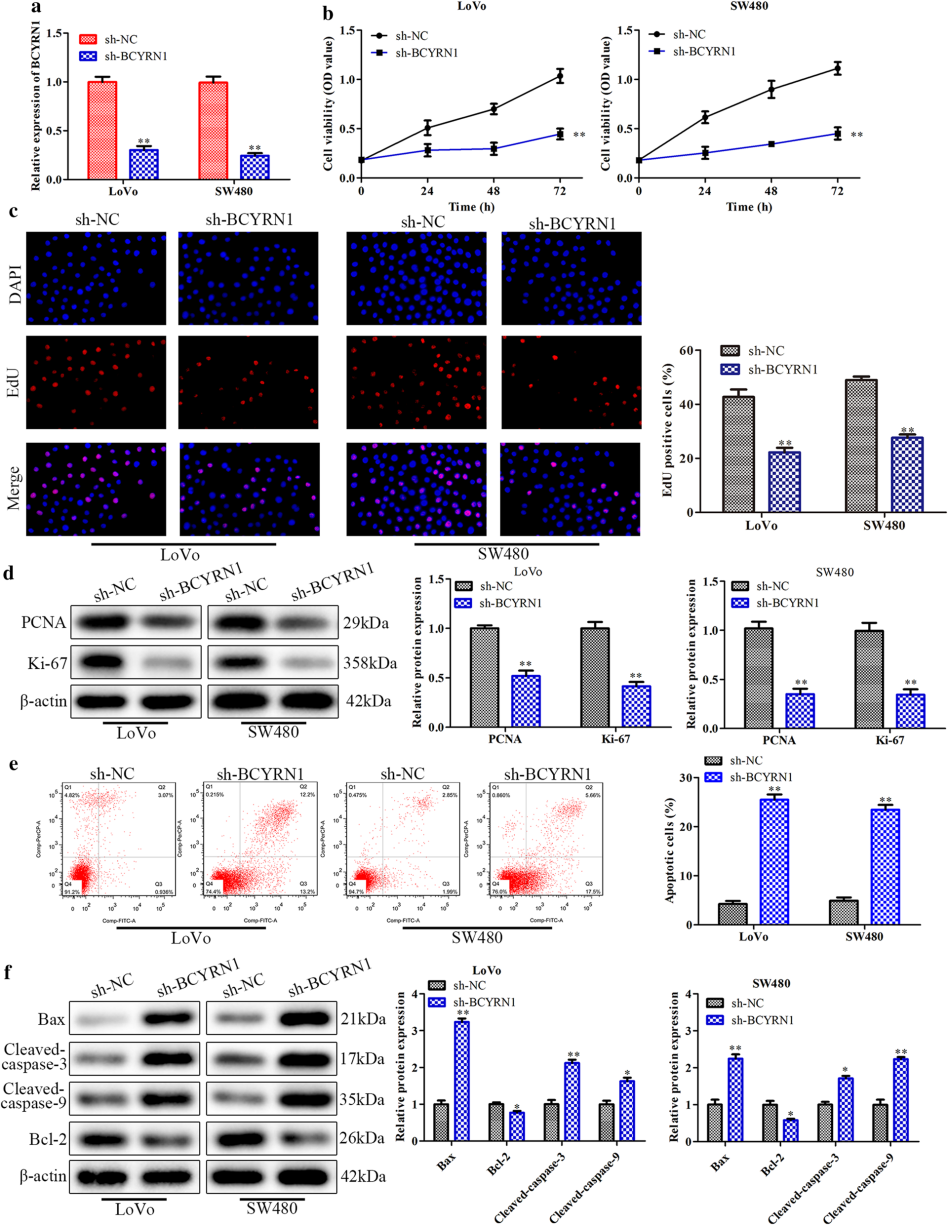
The effects of BCYRN1 knockdown on CRC cell proliferation and apoptosis. **a** BCYRN1 levels in LoVo and SW480 cells. **b** CRC cell proliferation was detected with CCK-8 assay. **c** The fluorescence images and data statistics chart of the EdU assay. **d** The expression of PCNA and Ki-67. **e** Original images and histogram of the flow cytometry assay. **f** The expression of apoptosis-related proteins in LoVo cells and SW480 cells. Values are the mean ± SD. *P < 0.05, **P < 0.01, and ***P < 0.001 vs. sh-NC. n = 3 per group

To further illuminate the roles of BCYRN1 in CRC, a series of experiments were designed in LoVo and SW480 cells. First, we used sh-BCYRN1 to downregulate endogenous BCYRN1, and the knockdown efficiency is shown in Fig. 2a. CCK-8 and EdU assays showed that downregulation of BCYRN1 evidently decreased the proliferation of LoVo and SW480 cells (Fig. 2b, c). Furthermore, knockdown of BCYRN1 obviously inhibited the expression of proliferation-related proteins, such as PCNA and Ki-67 (Fig. 2d). Subsequently, we detected cell apoptosis by flow cytometry analysis. The results showed that knockdown of BCYRN1 significantly increased apoptosis of LoVo and SW480 cells (Fig. 2e). In addition, several pro-apoptosis proteins, including Bax, cleaved caspase-3 and cleaved caspase-9 [32, 33], were highly expressed in LoVo and SW480 cells when they were treated with sh-BCYRN1. In contrast, the expression of Bcl-2, an inhibitor of cell apoptosis [33], was abolished in the sh-BCYRN1 group (Fig. 2f). These results indicated that BCYRN1 knockdown reduced proliferation while promoting the apoptosis of CRC cells.

### Downregulation of BCYRN1 decreases the migration and invasion of CRC cells

**Fig. 3:**
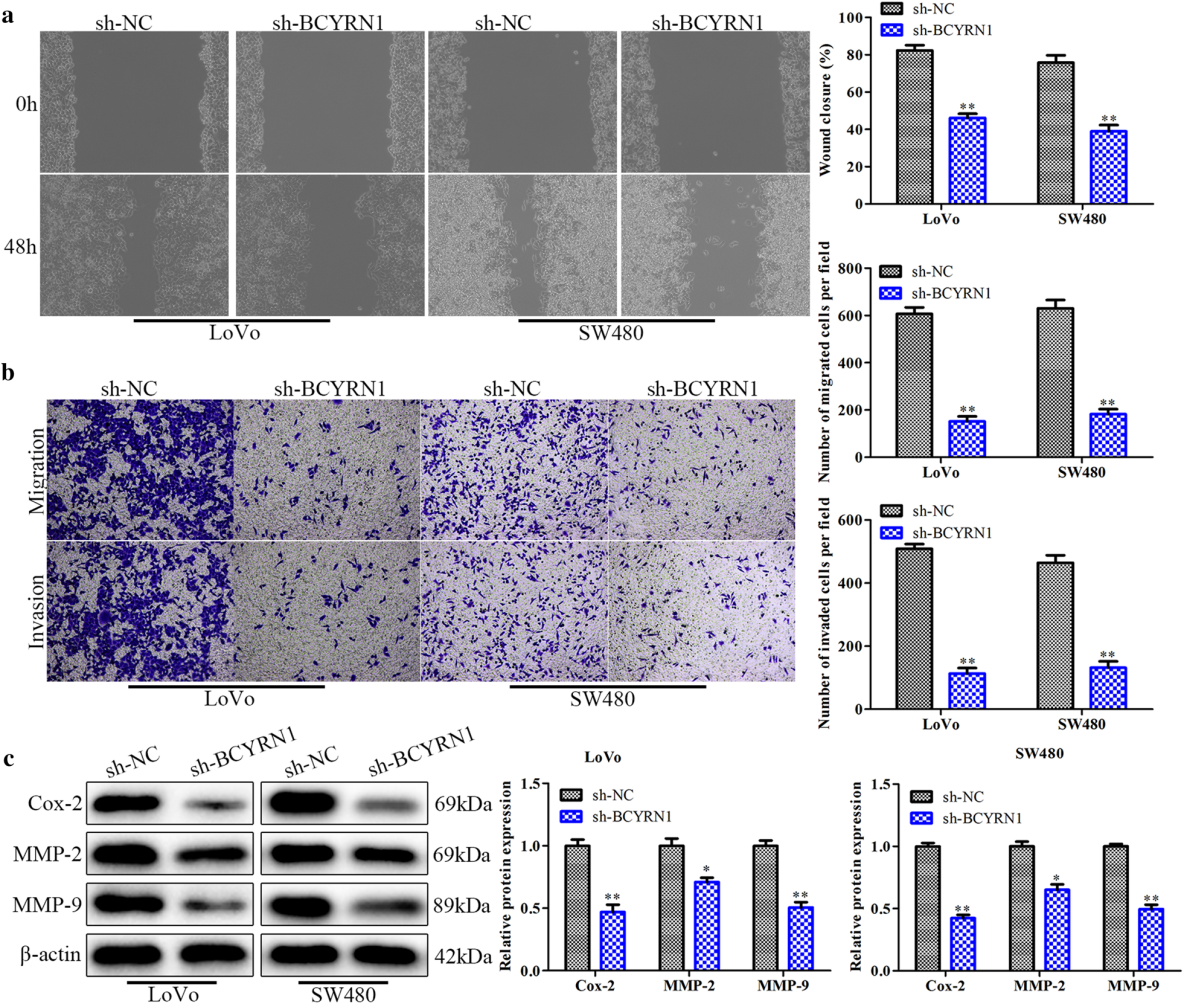
The effects of BCYRN1 knockdown on CRC cell migration and invasion. **a** The wound-healing assay showing the migration of CRC cells. **b** Images of the Transwell assay and histogram. **c** The expression of migration-related proteins determined by Western blot analysis. Values are the mean ± SD. *P < 0.05, **P < 0.01, and ***P < 0.001 vs. sh-NC. n = 3 per group

Next, we investigated the effects of BCYRN1 expression on CRC cell migration and invasion. Both wound healing and Transwell assays revealed that the migration and invasion of LoVo and SW480 cells were effectively suppressed by sh-BCYRN1 transfection (Fig. 3a, b). Consistently, migration- and invasion-related proteins, such as Cox-2, MMP-2 and MMP-9, were inhibited in LoVo and SW480 cells treated with sh-BCYRN1 (Fig. 3c). These results elucidated that BCYRN1 levels were closely related to the migration and invasion properties of CRC cells.

### miR-204-3p is a target gene of BCYRN1

**Fig. 4:**
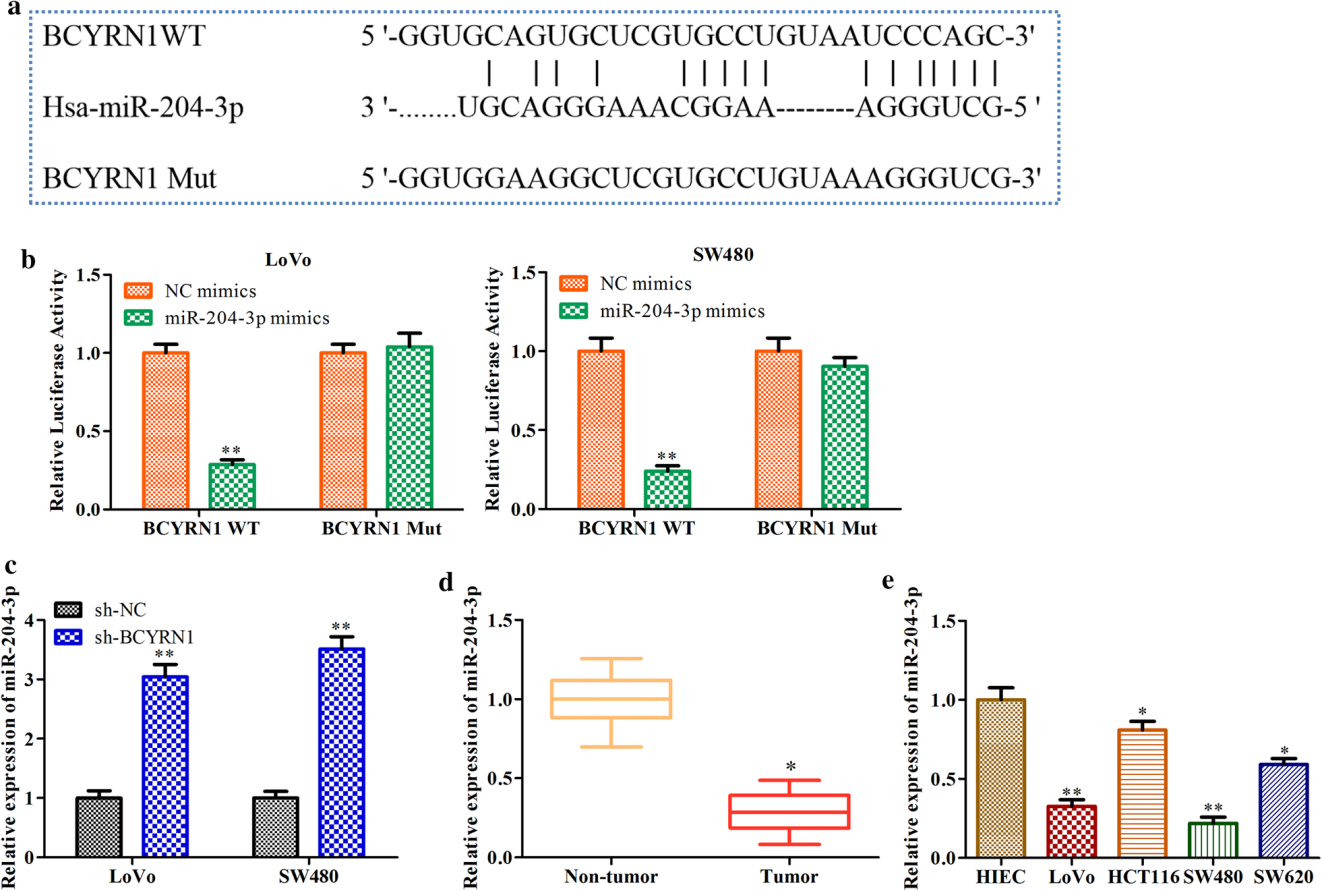
The target relationship between BCYRN1 and miR-204-3p. **a** The predicted location of the miR-204-3p combination according to LncBase Predicted V.2 analysis. **b** The results of the dual luciferase reporter assay. **c** The effects of BCYRN1 knockdown on miR-204-3p expression in LoVo and SW480 cells. **d** miR-204-3p levels in CRC tumour tissues and HC tissues. Data from 15 HC people and 15 GC patients. **e** miR-204-3p levels in HIECs and CRC cell lines. Values are the mean ± SD. *P < 0.05, **P < 0.01, and ***P < 0.001 vs. NC mimics, HC group, HIECs group, or sh-NC. n = 3 per group

A recent report showed that lncRNAs function as ceRNAs to competitively bind to miRNAs [11]. To illuminate the potential regulatory mechanism through which BCYRN1 affects CRC progression, we performed bioinformatic analysis with LncBase Predicted V.2 and found that BCYRN1 possesses a putative binding site for miR-204-3p (Fig. 4a). We further retrieved potential targets of BCYRN1 in different databases. To determine the interaction of BCYRN1 and miR-204-3p, we performed dual luciferase reporter analysis and found a significant reduction in the luciferase activity of BCYRN1 WT when LoVo or SW480 cells were transfected with miR-204-3p mimics. However, there was no notable difference in the luciferase activity of BCYRN1 Mut between the miR-204-3p mimic group and the negative control (NC) group (Fig. 4b). Accordingly, downregulation of BCYRN1 distinctly increased the expression of miR-204-3p in LoVo and SW480 cells (Fig. 4c). In addition, qRT-PCR results revealed that miR-204-3p was significantly reduced in CRC tumour tissues and cell lines, especially in LoVo and SW480 cells (Fig. 4d, e). These data proved that miR-204-3p is a target gene of BCYRN1 and is negatively regulated by BCYRN1.

### Effects of miR-204-3p on CRC cell proliferation, migration, invasion and apoptosis

**Fig. 5:**
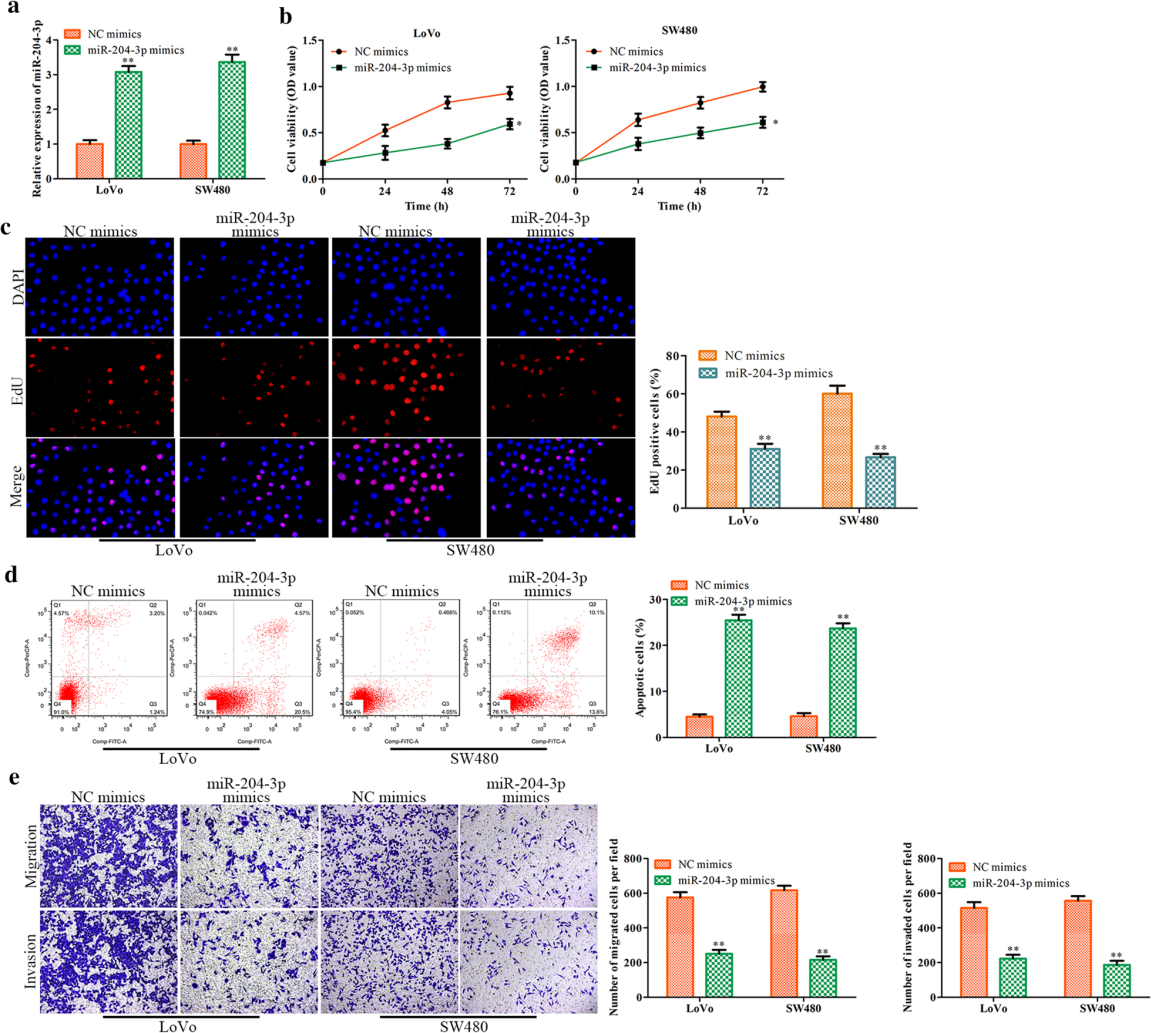
The effects of miR-204-3p overexpression on CRC cell proliferation, migration, invasion and apoptosis. **a** miR-204-3p expression in LoVo and SW480 cells. **b** CRC cell proliferation was evaluated with CCK-8 assay and EdU assay (**c**). **d** LoVo and SW480 cell apoptosis was determined with flow cytometry assay. **e** Original images and histogram of the Transwell assay. Values are the mean ± SD. *P < 0.05, **P < 0.01, and ***P < 0.001 vs. NC mimics group. n = 3 per group

We then studied the role of miR-204-3p in the regulation of CRC progression. First, we used miR-204-3p mimics to treat LoVo and SW480 cells. The overexpression efficiency of miR-204-3p is shown in Fig. 5a. Then, we found that miR-204-3p upregulation apparently inhibited cell proliferation, as demonstrated by CCK-8 and EdU assays (Fig. 5b, c). In addition, flow cytometry analysis revealed that miR-204-3p overexpression accelerated cell apoptosis (Fig. 5d). Consistently, CRC cell migration was obviously decreased by miR-204-3p mimics (Fig. 5e). These results concluded that miR-204-3p upregulation inhibits CRC cell proliferation, migration and invasion while promoting CRC cell apoptosis.

### KRAS is a target gene of miR-204-3p

**Fig. 6:**
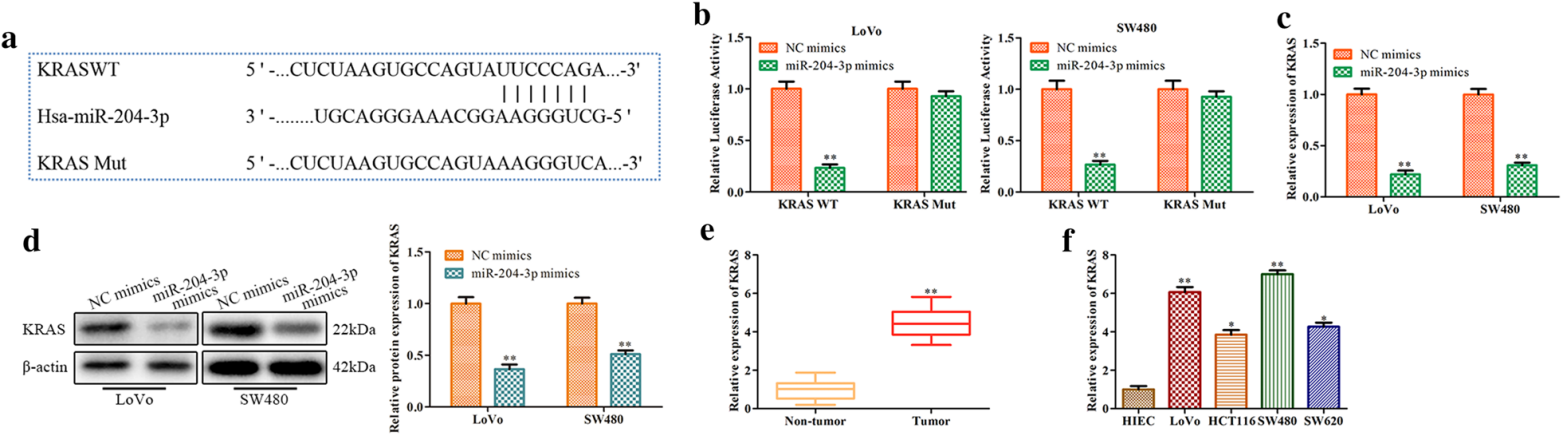
KRAS is a target gene of miR-204-3p. **a** The predicted location of the KRAS combination according to TargetScan. **b** The results of the dual luciferase reporter assay. **c**, **d** The effects of miR-204-3p overexpression on KRAS mRNA and protein expression in LoVo and SW480 cells. **e** KRAS mRNA levels in tumour tissues and HC tissues. Data from 15 HCs and 15 GC patients. **f** KRAS mRNA levels in HIECs and CRC cell lines. Values are the mean ± SD. *P < 0.05, **P < 0.01, and ***P < 0.001 vs. NC mimics group, HC group or HIECs group. n = 3 per group

We further explored the underlying mechanism of miR-204-3p in CRC. TargetScan, a miRNA target prediction database, was used to predict the downstream gene of miR-204-3p. We found that KRAS was a possible target gene of miR-204-3p, and the predicted position of the combination was in the 310–317 region of the KRAS 3′-UTR (Fig. 6a). It is generally known that KRAS is a crucial carcinogenic factor [34]. Moreover, the results of dual luciferase reporter analysis confirmed that KRAS was indeed the target gene of miR-204-3p (Fig. 6b). More significantly, overexpression of miR-204-3p inhibited the expression of KRAS mRNA and protein in LoVo and SW480 cells (Fig. 6c, d). We next investigated the relationship between KRAS levels and CRC progression. We noted that KRAS was highly expressed in CRC tumour tissues and cell lines (Fig. 6e, f). These results indicated that KRAS is a target gene of miR-204-3p and is negatively regulated by miR-204-3p.

### Effects of the BCYRN1/miR-204-3p/KRAS axis on CRC cell proliferation, migration, invasion and apoptosis

**Fig. 7:**
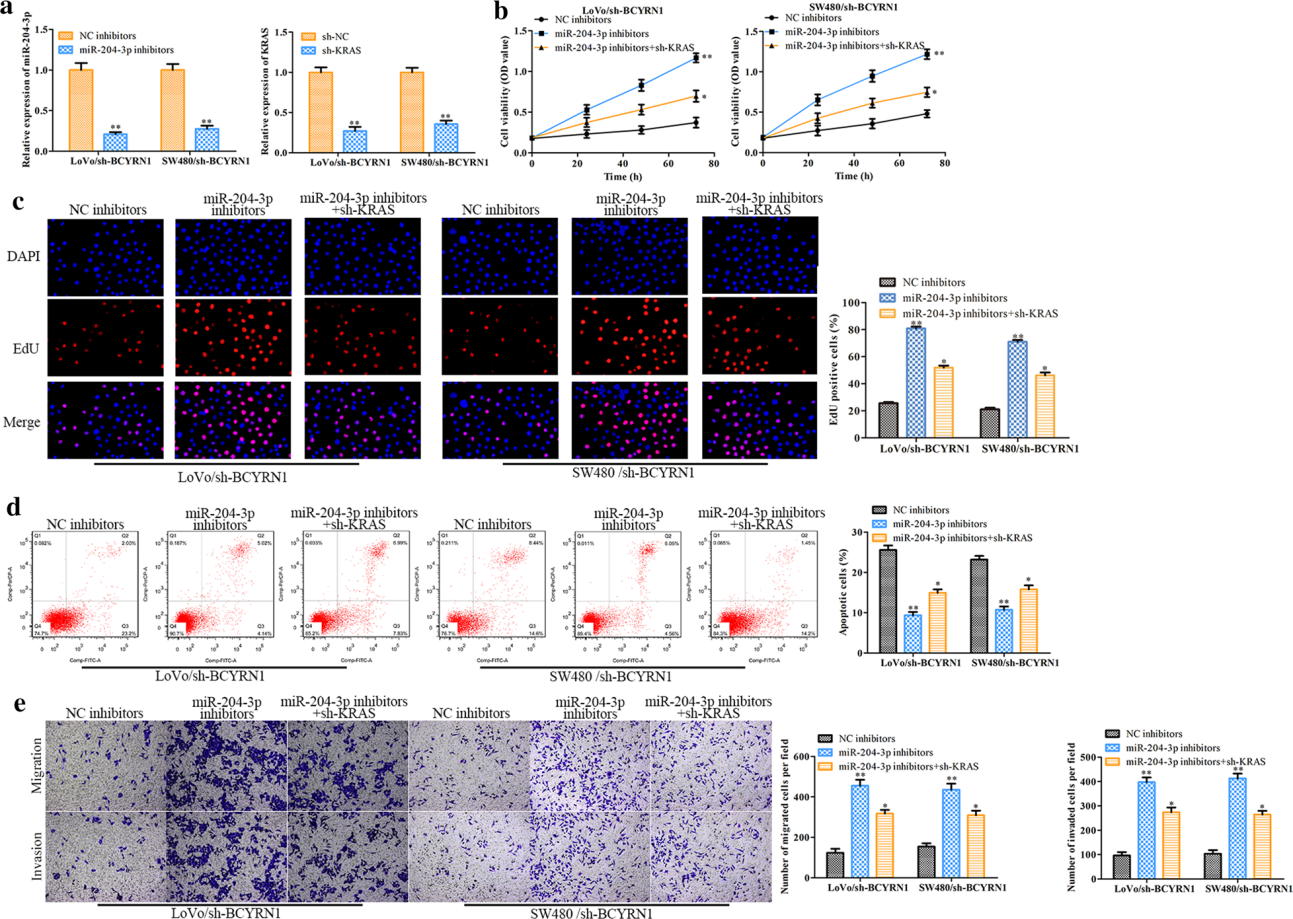
The effects of the BCYRN1/miR-204-3p/KRAS axis on CRC progression in vitro. **a** miR-204-3p expression in LoVo and SW480 cells with BCYRN1 knockdown. **b**, **c** The effects of proliferation determined by CCK-8 assay and EdU analysis. **d** Original images and histogram of the flow cytometry assay. **e** Cell migration detected by Transwell assay. Values are the mean ± SD. *P < 0.05, **P < 0.01, and ***P < 0.001 vs. NC inhibitor group. ^#^*P* < 0.05, ^##^*P* < 0.01, and ^###^*P* < 0.001 vs. the miR-204-3p inhibitor group. n = 6 per group

To understand the effects of the BCYRN1/miR-204-3p/KRAS axis on CRC progression, we designed a series of rescue experiments using miR-204-3p inhibitor, sh-KRAS and sh-BCYRN1 in LoVo and SW480 cells. The knockdown efficiency of miR-204-3p and KRAS in CRC cells treated with sh-BCYRN1 is shown in Fig. 7a. Then, we found that the miR-204-3p inhibitor significantly facilitated the proliferation of LoVo and SW480 cells treated with sh-BCYRN1, whereas sh-KRAS effectively reversed these effects (Fig. 7b, c). Furthermore, the miR-204-3p inhibitor abolished the apoptosis of sh-BCYRN1-treated CRC cells, whereas sh-KRAS relieved these phenomena (Fig. 7d). In addition, the migration of sh-BCYRN1-treated CRC cells was evidently increased in the miR-204-3p inhibitor-treated group, while sh-KRAS reversed these roles (Fig. 7e). These data indicated that BCYRN1 worsens CRC progression at least in part by inhibiting miR-204-3p levels and promoting KRAS expression.

### Knockdown of BCYRN1 inhibits tumour growth

**Fig. 8:**
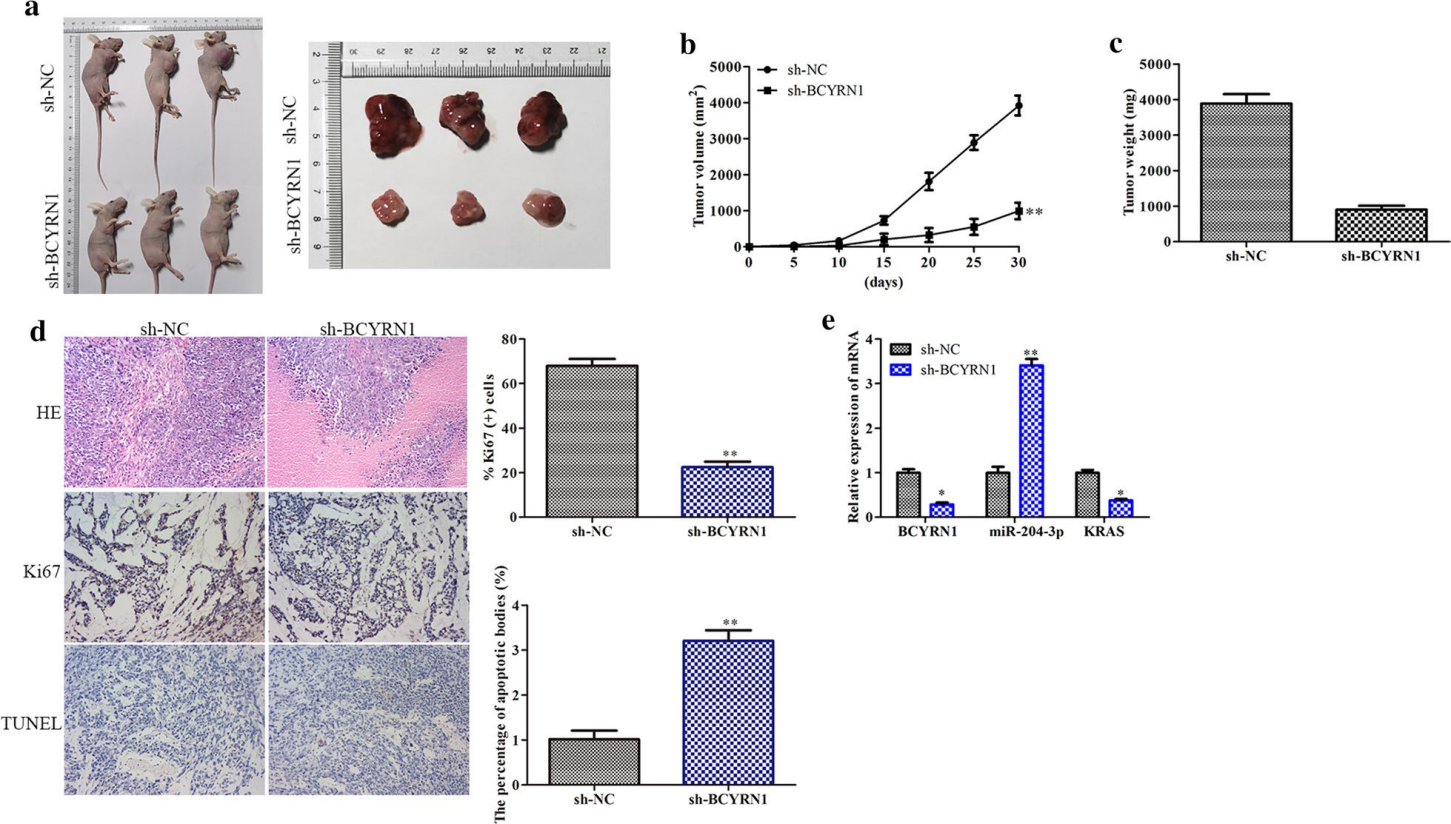
Effects of BCYRN1 knockdown on tumour development in vivo. **a** Phenotype images of tumours. **b** The line graph of tumour volume. **c** The bar chart of tumour weight. **d** Representative images and histograms of HE staining, immunohistochemical staining and TUNEL fluorescence staining. **e** RNA expression levels of BCYRN1, miR-204-3p and KRAS in mouse tumour tissues. Values are the mean ± SD. *P < 0.05, **P < 0.01, and ***P < 0.001 vs. sh-NC. n = 3 per group

To evaluate the therapeutic potential of BCYRN1 in CRC patients, we performed BCYRN1 knockdown by using sh-BCYRN1 in CRC model mice. Encouragingly, knockdown of BCYRN1 significantly decreased the tumour volume and weight of CRC model mice (Fig. 8a–c). HE staining, immunohistochemical staining and TUNEL staining showed that downregulation of BCYRN1 inhibited Ki-67 expression and promoted cancer cell apoptosis (Fig. 8d). More importantly, sh-BCYRN1 treatment obviously promoted the expression of miR-204-3p and decreased the expression of KRAS (Fig. 8e). These results revealed that knockdown of BCYRN1 inhibited the development of CRC.

## Discussion

Cancer mortality remains an enormous public health challenge on a global scale [35]. Although new advances have been made in the genomic changes in most different cancers [36], the molecular mechanisms underlying the development of CRC are poorly understood. Moreover, compared with patients with other malignancies, a large number of CRC patients have distant metastasis at the early stage of diagnosis, which leads to a low 5-year survival rate of approximately 10%-20% [37]. In addition, therapeutic methods are very limited in advanced CRC patients and mainly involve combinations of toxic drugs, such as 5-fluorouracil, irinotecan and/or oxaliplatin, for chemotherapy. Therefore, the molecular mechanism involved in the occurrence and development of CRC needs to be explored urgently. Our current study revealed an important new mechanism of the occurrence of CRC and provided a novel idea for the effective treatment of CRC, briefly, to control CRC progression by blocking BCYRN1.

LncRNAs have been shown to play vital roles in the regulation of cancer progression [13, 14], and the main functions of lncRNAs are recruiting chromatin modification complexes for transcriptional regulation [38] and interacting with miRNAs, mRNAs, and/or proteins for posttranscriptional regulation [11, 12]. It has been proven that one of most common and crucial roles of lncRNA is their functions as a “miRNA sponges” [27, 39]. This means that lncRNA can be used as a bait to bind with a specific miRNA, resulting in the inability of the miRNA to combine with its corresponding mRNA. BCYRN1 is a newly identified lncRNA with similar characteristics [15]. Previous studies have revealed that miRNAs, such as miR-125-5p, miR-149 and miR-490-3p, act as targets of BCYRN1 in hepatocellular carcinoma, human glioma and lung cancer, respectively [17, 40, 41]. Recently, the relationship between BCYRN1 and CRC has been gradually observed. Gu et al. first found that BCYRN1 levels were closely related to the occurrence, development and prognosis of CRC, and microarray bioinformatics analysis confirmed that the possible target of BCYRN1 was the NRP3 gene [42]. Moreover, Yu JH and Chen Y proved that BCYRN1 levels were significantly increased in tumour tissues and cell lines (SW620) of CRC by a series of well-designed in vitro experiments [43]. However, it remains obscure whether BCYRN1 can control miRNAs to regulate the progression of CRC. Our present study indicated that BCYRN1 is involved in the occurrence and development of CRC through specific binding to a new target miRNA: miR-204-3p. We found that BCYRN1 was highly expressed, while the expression levels of miR-204-3p were significantly reduced in the tissues and cell lines of CRC compared to those of controls. In addition, knockdown of BCYRN1 distinctly increased miR-204-3p expression. These findings provide new insights into the mechanism of CRC progression and the treatment of CRC.

As mentioned above, miRNAs are involved in the regulation of cancer [22, 23]. Interestingly, miRNAs can act as tumour suppressor genes [44] or oncogenes [45] to regulate the biological characteristics of cancer. Cui et al. reported that miR-204-3p acts on its potential target gene fibronectin 1 (FN1) and inhibits its expression, thereby decreasing the growth of hepatocellular carcinoma tumour endothelial cells [46]. Recently, several studies have reported the roles of miR-204-3p in ovarian cancer [47], lung adenocarcinoma [48] and bladder cancer [49], but the functions of miR-204-3p in CRC remain obscure. Our current research showed that miR-204-3p is a potential target gene of BCYRN1 and is negatively regulated by BCYRN1. We further investigated whether miR-204-3p overexpression inhibited CRC cell proliferation, migration and invasion while exacerbating CRC cell apoptosis. In addition, bioinformatics analysis and dual luciferase reporter assay proved that KRAS was a potential target gene of miR-204-3p. KRAS is an important oncogene and a key tumour maintenance gene in many carcinomas [34]. Then, we showed that KRAS was highly expressed in CRC tissues and cell lines, and overexpression of miR-204-3p eliminated this effect. These results indicated that overexpression of miR-204-3p and downregulation of KRAS can be considered new approaches to the treatment of CRC.

In summary, these findings demonstrated that BCYRN1 participates in the occurrence and development of CRC. In terms of functional regulation, BCYRN1 promotes CRC progression at least in part by regulating the miR-204-3p/KRAS axis. Our study broadens the current understanding of BCYRN1 function in CRC and provides a novel therapeutic target for CRC.

## Conclusion

1. BCYRN1 is highly expressed in CRC tissues and cell lines compared to normal controls.
2. Downregulation of BCYRN1 decreases the migration and invasion of CRC cells. Knockdown of BCYRN1 promotes the apoptosis of CRC cells.
3. miR-204-3p is a target gene of BCYRN1 and affects CRC cell proliferation, migration, invasion and apoptosis.
4. KRAS is a target gene of miR-204-3p, and the BCYRN1/miR-204-3p/KRAS axis affects the proliferation, migration, invasion and apoptosis of CRC cells.
5. Knockdown of BCYRN1 inhibits tumour growth.

## Data Availability

Yes.
